# The neural correlates of risk propensity in males and females using resting-state fMRI

**DOI:** 10.3389/fnbeh.2014.00002

**Published:** 2014-01-28

**Authors:** Yuan Zhou, Shu Li, John Dunn, Huandong Li, Wen Qin, Maohu Zhu, Li-Lin Rao, Ming Song, Chunshui Yu, Tianzi Jiang

**Affiliations:** ^1^Key Laboratory of Behavioral Science, Institute of Psychology, Chinese Academy of SciencesBeijing, China; ^2^The School of Psychology, The University of AdelaideAdelaide, SA, Australia; ^3^Brainnetome Center and National Laboratory of Pattern Recognition, Institute of Automation, Chinese Academy of SciencesBeijing, China; ^4^Department of Radiology, Tianjin Medical University General HospitalTianjin, China; ^5^Key Laboratory for NeuroInformation of Ministry of Education, School of Life Science and Technology, University of Electronic Science and Technology of ChinaChengdu, China; ^6^The Queensland Brain Institute, The University of QueenslandBrisbane, QLD, Australia

**Keywords:** functional connectivity, functional magnetic resonance imaging, resting state, risk propensity, sex difference

## Abstract

Men are more risk prone than women, but the underlying basis remains unclear. To investigate this question, we developed a trait-like measure of risk propensity which we correlated with resting-state functional connectivity to identify sex differences. Specifically, we used short- and long-range functional connectivity densities to identify associated brain regions and examined their functional connectivities in resting-state functional magnetic resonance imaging (fMRI) data collected from a large sample of healthy young volunteers. We found that men had a higher level of general risk propensity (GRP) than women. At the neural level, although they shared a common neural correlate of GRP in a network centered at the right inferior frontal gyrus, men and women differed in a network centered at the right secondary somatosensory cortex, which included the bilateral dorsal anterior/middle insular cortices and the dorsal anterior cingulate cortex. In addition, men and women differed in a local network centered at the left inferior orbitofrontal cortex. Most of the regions identified by this resting-state fMRI study have been previously implicated in risk processing when people make risky decisions. This study provides a new perspective on the brain-behavioral relationships in risky decision making and contributes to our understanding of sex differences in risk propensity.

## Introduction

Most decisions involve risk, and the general willingness to take such risks is called risk propensity (Harrison et al., 2005). Men tend to have higher levels of risk propensity than women (Byrnes et al., 1999; Croson and Gneezy, 2009; Wang et al., 2009; Dohmen et al., 2011), a difference that is consistent across time and in a variety of contexts (Powell and Ansic, 1997). Despite these well-documented sex differences, little is known regarding their neural correlates. Two previous studies have examined the activation of brain regions during a gambling task and risk-taking task but found inconsistent patterns of sex differences (Bolla et al., 2004; Lee et al., 2009). In one of these studies, task-reduced activity in the insula and right lateral orbitofrontal cortex (OFC) was associated with risk preference in women, but not in men (Lee et al., 2009). However, these studies as well as others which did not examine sex-related differences (e.g., Paulus et al., 2003; Hsu et al., 2005; Tobler et al., 2007; Christopoulos et al., 2009), investigated changes in brain activity associated with risky decision making tasks and so did not directly address the neural correlates of the enduring trait of risk propensity. How the neural correlates of this general disposition are differentially expressed in men and women is currently unknown. To address this question, it is necessary to find a stable measure of risk propensity and to correlate it with a similarly stable and enduring characteristic of neural activity.

Resting-state functional magnetic resonance imaging (fMRI) is an important approach in the study of human brain functions (Raichle, 2010). Resting-state functional connectivity (RSFC), which measures inter-regional correlations among spontaneous low-frequency (<0.1 Hz) fluctuations in the fMRI signal (Biswal et al., 1995), can be used to identify enduring and intrinsic properties of the brain (Fox and Raichle, 2007). As well as reflecting underlying anatomical connectivity (Park and Friston, 2013), RSFC has also been shown to correspond to the brain's functional architecture in response to external stimuli (Raichle and Mintun, 2006; Smith et al., 2009), as evidenced by findings that intrinsic resting-state brain activity can predict task-evoked brain activation during different cognitive tasks (Fox et al., 2006, 2007; Mennes et al., 2010; Liu et al., 2011; Mennes et al., 2011; Zou et al., 2013). RSFC has also been used to characterize functional brain networks correlated with individual differences in behavioral traits, such as personality, autistic trait and aggression (Di Martino et al., 2009; Hoptman et al., 2010; Adelstein et al., 2011). To our knowledge, only two studies have explored the neural correlates of risk propensity using RSFC (Cox et al., 2010; Han et al., 2012). Using a seed-based RSFC approach, these groups have associated the RSFCs of brain regions implicated in the evaluation of risk and reward (nucleus accumbens) as well as cognitive control and risk aversion (anterior insula and anterior cingulate cortex) with various risk-aversion measures. Both studies have suggested that individual differences in risk propensity are correlated with the brain's intrinsic functional architecture, which, in turn, may reflect experience-based recruitment of specific brain regions in the active processing of risk. However, these studies did not address the neural correlates of sex differences in risk propensity, and the seed-based approach employed limited their findings to specific regions.

To detect functional networks underlying sex differences in risk propensity, we used a functional network analysis based on RSFC. Functional connectivity density mapping (FCDM) is a recently proposed voxel-wise and model-free approach to the measurement of RSFC (Tomasi and Volkow, 2010, 2012b,d). It can be used to identify brain regions correlated with the measure of interest as well as to reveal details of functional networks centered on these regions when combined with seed-based RSFC (Tomasi and Volkow, 2012a). Accordingly, in this large-sample resting-state fMRI study, we developed a composite measure of the trait of general risk propensity (GRP) and examined its neural correlates in men and women using a whole brain search strategy based on FCDM combined with seed-based RSFC (Tomasi and Volkow, 2012a). We hypothesized that the neural correlates of GRP in men possibly differ from that in women in the resting-state functional networks centered at the regions previously implicated in sex-related differences in risk-taking behaviors (Bolla et al., 2004; Lee et al., 2009), consistent with the view that RSFC reflects intrinsic representations of the brain's functional repertoire (Smith et al., 2009). In addition, we predicted that men and women may share the same neural correlates of GRP in resting-state functional networks centered at the regions supporting cognitive control, such as inferior frontal cortex (Aron et al., 2004), anterior insula or anterior cingulate cortex (Dosenbach et al., 2007, 2008), as indicated in previous studies regarding the RSFC of risk propensity (Cox et al., 2010; Han et al., 2012).

## Materials and methods

### Participants

Three hundred and twenty-four healthy right-handed participants were recruited by advertisement for the present study. Five participants were excluded due to missing behavioral data. Three additional participants were excluded due to raw imaging data errors, and 12 participants were excluded due to bad raw imaging data after two board-certified radiologists, Yu and Qin, manually inspected the raw fMRI data for all of the participants. Furthermore, 15 participants were excluded due to excessive head motion during the fMRI scan (see the following section). Thus, there were 289 healthy participants in the final group, consisting of 131 men (mean age = 22.2 years, *SD* = 2.5 years) and 158 women (mean age = 23.2 years, *SD* = 2.2 years). All of the participants gave written informed consent, and this study was approved by the ethical committee of the Tianjin Medical University General Hospital.

### Behavioral procedures

#### Assessment of risk propensity

A composite measure of risk propensity was developed from the following five scales, all of which are domain-free and stably measure different profiles of risk propensity. The scales used in this study were the Risk Propensity Scale (RPS), the Sensation Seeking Scale (SSS), the Eysenck Personality Questionnaire (EPQ), the Tridimensional Personality Questionnaire (TPQ), and the Barratt Impulsiveness Scale (BIS).

The RPS was developed to measure general risk-taking tendencies (Meertens and Lion, 2008). This is a short (7 items), practical, and easy-to-use scale that has been used in a number of previous experimental studies (Lion and Meertens, 2001, 2005).

The SSS Form V (Zuckerman et al., 1978) consists of 40 items related to four dimensions associated with sensation seeking: thrill and adventure seeking (SSS_TAS), the desire to engage in outdoor non-competitive sports or activities involving elements of risk; experience seeking (SSS_ES), the seeking of new experiences through the mind and senses and an unconventional style of life; disinhibition (SSS_DIS), particularly social disinhibition; and boredom susceptibility (SSS_BS), which is defined as the dislike of any repetition of experience, predictable, dull or boring people.

The EPQ (Eysenck, 1991) consists of 88 items that measure the personality traits: extraversion-introversion (EPQ_E), neuroticism or emotionality (EPQ_N), and psychotism (EPQ_P) or tough mindedness. And it also includes a lie subscale (EPQ_L). These constructs subsume elements of risk taking: impulsivity forms a part of the EPQ_P, and sensation seeking and venturesomeness form a part of the EPQ_E (Eysenck and Eysenck, 1985).

The TPQ (Cloninger et al., 1991) consists of 100 items that measure three dimensions of personality traits: novelty seeking (TPQ_NS), harm avoidance (TPQ_HA) and reward dependence (TPQ_RD). These traits have been previously used to predict risk taking behaviors, such as alcohol consumption, pathological gambling, substance abuse, and internet addiction (Kim and Grant, 2001; Hale et al., 2003; Skeel et al., 2008; Ko et al., 2010). Generally, persons with high novelty seeking, low harm avoidance, and low reward dependence are more likely to engage in these risky behaviors.

The BIS-11 (Patton et al., 1995) is a widely used self-reported measure of impulsive personality traits composed of three sub-scales: attentional (BIS_ATT), motor (BIS_MOT), and non-planning (BIS_NP) impulsiveness. Similar to sensation seeking, impulsiveness has been demonstrated as being driving forces of risk taking (Harrison et al., 2005). These traits have also been linked to both risk taking behaviors measured by experimental tasks (Lee et al., 2008; Cheng and Lee, 2012) and risk-taking behaviors in real life, such as risky driving behavior (Cheng and Lee, 2012).

#### Extraction of general risk propensity (GRP)

Because each of the five scales measures risky propensity in different ways, principle component analysis (PCA) was used to extract an index reflecting GRP. PCA is a general technique used to extract common or shared variance from a set of measures (Costello and Osborne, 2005) and has been previously used in similar contexts (Need et al., 2009).

The data were analyzed using SPSS v17.0. The item scores from the RPS and scores on the relevant subscales of the SSS, EPQ, TPQ, and BIS were used as variables. A total of 15 variables were entered into PCA with varimax rotation. The regression method was used to create the participant scores for each principal component. The score of the first rotated principal component was used as a measure of GRP in further analyses.

### MRI procedure

#### MR image acquisition

MR images were acquired on a 3.0 Tesla MR scanner (General Electric, Milwaukee, WI, USA). During scanning, foam paddings and earplugs were used to limit head motion and to reduce scanning noise. All of the participants received a three-dimensional magnetization prepared rapid acquisition gradient echo (3DMPRAGE) sequence (repetition time (*TR*)/echo time (*TE*) = 8.06/3.12 ms, flip angle = 13°, 176 sagittal slices, voxel size = 1 mm × 1 mm × 1 mm). The resting-state functional images were acquired using an echo-planar imaging (EPI) sequence that is sensitive to BOLD contrast with the following parameters: 40 slices, *TR*/*TE* = 2000/30 ms, thickness/gap = 4.0/0.0 mm, voxel size = 3.75 mm × 3.75 mm × 4.0 mm, FOV = 240 × 240 mm, matrix = 64 × 64, flip angle = 90°. Each functional run lasted for 6 min and contained 180 volumes. During the fMRI scans, all of the participants were instructed to keep their eyes closed, relax their minds, and move as little as possible.

#### Imaging data preprocessing and analyses

***Data preprocessing***. After inspection of the quality of the raw functional images, data preprocessing was performed using FSL tools (www.fmrib.ox.ac.uk/fsl) and AFNI (http://afni.nimh.nih.gov/afni). The following preprocessing steps were performed: discarding the first 10 slices for the magnetization equilibrium; slice timing correction; head motion correction; non-brain removal; temporal filtering (0.01–0.1 Hz) and regressing nuisance signals (6 motion parameters). Registration of the resting-state data to high-resolution T1-weighted images and the T1 non-linear data to a 3 mm isotropic MNI-152 standard space template (Montreal Neurological Institute) were performed. The resulting transformation matrices were combined to obtain a native MNI space transformation matrix and its inverse. Participants who had a maximum displacement greater than 2 mm in any of the cardinal directions (*x, y, z*), and a maximum spin (*x, y, z*) greater than 2° were excluded from subsequent analysis.

***Short- and long-range FCD***. FCDM was used to characterize individual functional connectivity maps with high spatial resolution (≥3 mm isotropic) (Tomasi and Volkow, 2010). This method is free from the constraints of a priori selection of specific seed regions and allows the identification and location of functional hubs in a whole brain network from the perspectives of short- and long-range FCD (Tomasi and Volkow, 2012b). In the application of this method, a global FCD was first computed. On the basis of previous work, two voxels were considered functionally connected if their Pearson correlation coefficient was greater than 0.6 (Tomasi and Volkow, 2012b). The global FCD at a given voxel was defined as the number of functional connections between this voxel and all other voxels in the brain. Next, we used a “growing” algorithm developed in C to derive the short-range FCD. In this algorithm, for a given voxel, *x*_0_, an additional voxel, *x_j_* was added to the list of neighbors of *x*_0_ if it was adjacent to a voxel that was linked to *x*_0_ by a continuous path of functionally connected voxels and the correlation coefficient between *x*_0_ and *x*_*j*_ was greater than 0.6. This calculation was repeated for all of the voxels that were adjacent to the neighbors of *x*_0_ in an iterative manner until no new neighbors could be added to the list. The short-range FCD of *x*_0_ was defined as the number of elements in the list of neighbors. The strength of the long-range FCD was equal to the difference between the global FCD and short-range FCD. These calculations were performed on all of the voxels in the brain. Thus, the strength of the short-range FCD in a voxel reflects the functional correlation between this voxel and other voxels within a local cluster, and the strength of the long-range FCD reflects the functional correlation between this voxel and other voxels located at a distance. Finally, the short- and long-range FCD maps were spatially smoothed using a Gaussian kernel of full-width at half-maximum (FWHM) of 8 mm. For standardization purposes, the short- and long-range FCD of each voxel was divided by the global mean short- and long-range FCD, respectively.

***RSFC networks***. To reveal the specific networks associated with the GRP, the regions selected on the basis of the results of the short- and long-range FCD analyses were used as seed regions for the seed-based RSFC. The mean time series of each seed region were acquired by averaging the time series of all of the voxels within that region. Pearson's correlation coefficients were then computed between the mean time series of the seed region and the time series for each voxel in the brain. The correlation coefficients were then converted into *z*-values using Fisher's r-to-z transformation to improve their normality. Finally, an individual RSFC map was obtained.

### Statistical analyses

Using SPM8, the normalized short- and long-range FCD maps computed for each participant were regressed on the variables of GRP, sex, and the GRP by sex interaction. The statistical significance was determined by Monte Carlo simulations to obtain a *p*-corrected < 0.05 after correcting for whole brain comparisons. The corrected threshold corresponds to *p*-uncorrected < 0.001 with a minimum cluster size of 513 mm^3^ (a gray mask with 55,342 voxels was used).

To assess the significance of the findings in a cluster of voxels rather than in a single voxel, the average strengths of the normalized short- and long-range FCD in regions with significant main or interaction effects were extracted from individual maps. These measures were then analyzed using SPSS v17.0. If an interaction effect was statistically significant, then *post-hoc* testing was performed using MODPROBE, which is an aid used to probe single-degree-of-freedom interactions in OLS and logistic regression analyses (Hayes and Matthes, 2009).

Similar statistical analyses were applied to the RSFC maps. In this study, the RSFC map computed for each participant was regressed on the GRP, sex and sex by GRP. The whole brain comparisons were constrained by a mask composed of a set of regions showing significant connectivities with the seed region. The statistical significance was determined by Monte Carlo simulations to obtain the *p*-corrected < 0.05. The corrected threshold corresponds to a *p*-uncorrected < 0.005 with a minimum cluster size of 999 mm^3^ (which is dependent on the size of each mask).

## Results

### Behavioral measure of the GRP

The 15 measures were used to assess the GRP (for the mean and standard deviation of each measure, please see Table A1 in the Appendix). The variables correlated moderately well with each other, with no particularly large correlations (the largest absolute value of the correlation coefficient was 0.63). The Kaiser-Meyer-Olkin value was 0.748, and the Bartlett's test results were significant (approx. Chi-Square = 1179.2, *p* < 0.001), indicating that PCA was appropriate. This revealed four components with eigenvalues greater than 1. The loadings of each variable for each component after the rotation are shown in Table 1. Component 1 accounted for the most variance among the four components (21.8, 18.0, 8.7, and 7.3%, respectively). The variables that loaded strongly on to Component 1 were those that were related to general risk-taking tendency, novelty/sensation seeking, venturesomeness and harm avoidance. For this reason, we identified Component 1 as reflecting GRP. High scores of GRP indicated risk taking and lower GRP scores indicated risk aversion.

**Table 1:** **Loadings for principal components using varimax rotation**.

**Roation Variables**	**Component**			
	**1 (Risk-seeking)**	**2 (Impulsivity)**	**3 (Lack of control)**	**4 (Reward-dependence)**
TPQ_HA	−0.774			
EPQ_E	0.662			0.543
SSS_ES	0.599			
EPQ_N	−0.578		0.546	
SSS_DIS	0.531			
RPS	0.487			
SSS_TAS	0.486			
BIS_MOT		0.814		
TPQ_NS	0.354	0.657		
BIS_NP		0.652		−0.319
BIS_ATT		0.525	0.421	
EPQ_P			0.699	−0.320
SSS_BS			0.670	
EPQ_L			−0.596	
TPQ_RD				0.824

*Abbreviations: BIS_ATT, attentional factors of the Barratt Impulsiveness Scale; BIS_MOT, motor factors of the Barratt Impulsiveness Scale; BIS_NP, non-planning impulsiveness factors of the Barratt Impulsiveness Scale; EPQ_E, extraversion-introversion subscale of the Eysenck Personality Questionnaire; EPQ_L, lie subscale; EPQ_N, neuroticism subscale of the Eysenck Personality Questionnaire; EPQ_P, psychotism subscale of the Eysenck Personality Questionnaire; RPS, Risk Propensity Scale; SSS_TAS, thrill and adventure seeking dimention of the Sensation Seeking Scale; SSS_ES, experience seeking dimention of the Sensation Seeking Scale; SSS_DIS, disinhibition dimention of the Sensation Seeking Scale; SSS_BS, boredom susceptibility dimention of the Sensation Seeking Scale; TPQ_NS, novelty seeking subscale of the Tridimensional Personality Questionnaire; TPQ_HA, harm avoidance subscale of the Tridimensional Personality Questionnaire; TPQ_RD, reward dependence subscale of the Tridimensional Personality Questionnaire*.

Independent sample *t*-tests revealed that men had significantly higher GRP scores compared to women (mean/*SD* for men: 0.37/0.92; mean/*SD* for women: −0.31/0.95; *p* < 0.001).

### Short- and long-range functional connectivity

**Figure A1:**
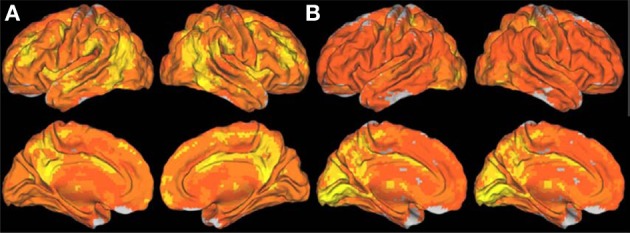
**Surface rendering showing the spatial distribution of the long- (A) and short-range (B) FCD in the human brain, which reflect the average number of functional connections per voxel across 289 participants**. The images were created using the Computerized Anatomical Reconstruction and Editing Toolkit (CARET) 5.62, which is a free software developed at Washington University (http://brainvis.wustl.edu/wiki/index.php/Caret:About).

The spatial distribution of the short-range FCD was highly localized in the posterior cingulate/ventral precuneus, occipital (cuneus and calcarine cortex) regions, middle cingulate cortex, anterior cingulate cortex/medial prefrontal cortices, the inferior parietal regions, thalamus and striatum. This distribution was similar to those previously reported by Tomasi and Volkow (2010, 2012d). The spatial distribution of the long-range FCD was also localized in the abovementioned regions with the maxima in the posterior cingulate/ventral precuneus and medial prefrontal cortices. Bilateral dorsolateral prefrontal, insula, lateral parietal regions and temporal cortices also had high long-range FCDs (Figure A1 in Appendix).

#### General risk propensity

**Figure 1:**
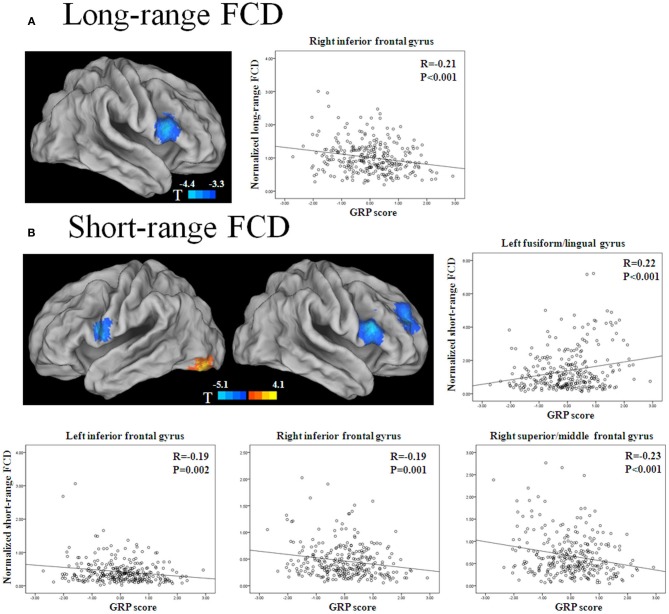
**Statistical significance (*T*-score) of the correlation with the GRP score for long-range FCD (A) and short-range FCD (B) across 289 healthy participants, overlaid on the surface of the Colin template**. The cool color represents a negative correlation, and the warm color represents a positive correlation. The images were created using the Computerized Anatomical Reconstruction and Editing Toolkit (CARET) 5.62, which is a free software that was developed at Washington University (http://brainvis.wustl.edu/wiki/index.php/Caret:About). Scatter plots show GRP-related changes in long- or short-range FCD. The lines are linear fits of the data.

The long-range FCD in the right inferior frontal gyrus (IFG) decreased with the GRP score (*p* < 0.05, corrected) (Figure 1A, Table 2). The short-range FCD in the bilateral inferior frontal gyri and right dorsolateral prefrontal cortex also decreased with the GRP score, while that in the left fusiform gyri extending to the lingual gyri increased with the GRP score (Figure 1B, Table 2).

**Table 2:** **Main effect of the general risk propensity scores on long- and short-range FCD**.

**Cluster size**	**Hemisphere**	**Brain region**	**BA**	**MNI coordinates**			**Peak *F*-value**
**LONG-RANGE FCD**							
93	Right	Inferior frontal gyrus	44/45	51	21	18	19.33
**SHORT-RANGE FCD**							
119	Right	Inferior frontal gyrus	45	54	21	15	25.58
90	Right	Middle frontal gyrus	10/9	27	54	33	19.2
59	Left	Fusiform gyrus/lingual gyrus	18/19	−33	−81	−15	16.52
39	Left	Inferior frontal gyrus	44	−54	6	15	15.42

*Abbreviations: BA, broadman area; FCD, functional connectivity density*.

#### Sex

**Figure A2:**
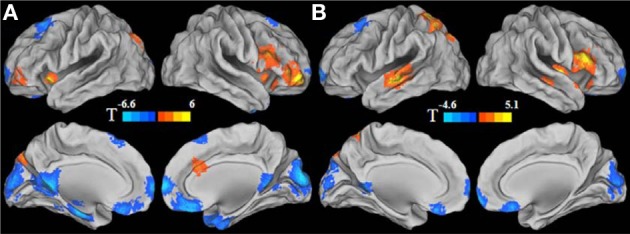
**Gender effect for long-range FCD (A) and for short-range FCD (B)**. For the purpose of presentation, cool color represents the regions showing reduced long- or short-range FCD in men, and warm color represents the regions showing reduced long- or short-range FCD in women. Statistical values were *T* values for the simple effects.

Strong sex differences for short- and long-range FCDs were found. Compared to men, women showed higher levels of long-range FCDs in the default mode network region (posterior cingulate cortex, medial frontal/orbitofrontal cortex, bilateral parahippocampal gyrus and its adjacent medial temporal cortices) (Greicius et al., 2003; Fox et al., 2005; Buckner et al., 2008), calcarine, superior frontal gyrus, cerebellum and pons, and showed lower levels of long-range FCDs in the bilateral ventral frontal and lateral orbitofrontal cortices, the bilateral insula, the right anterior/middle cingulate cortex and the left superior parietal lobe. The sex differences in short-range FCDs were similar, but more localized and weaker than the differences found in long-range FCDs (Figure A2 in Appendix).

#### GRP by sex interaction

**Figure 2:**
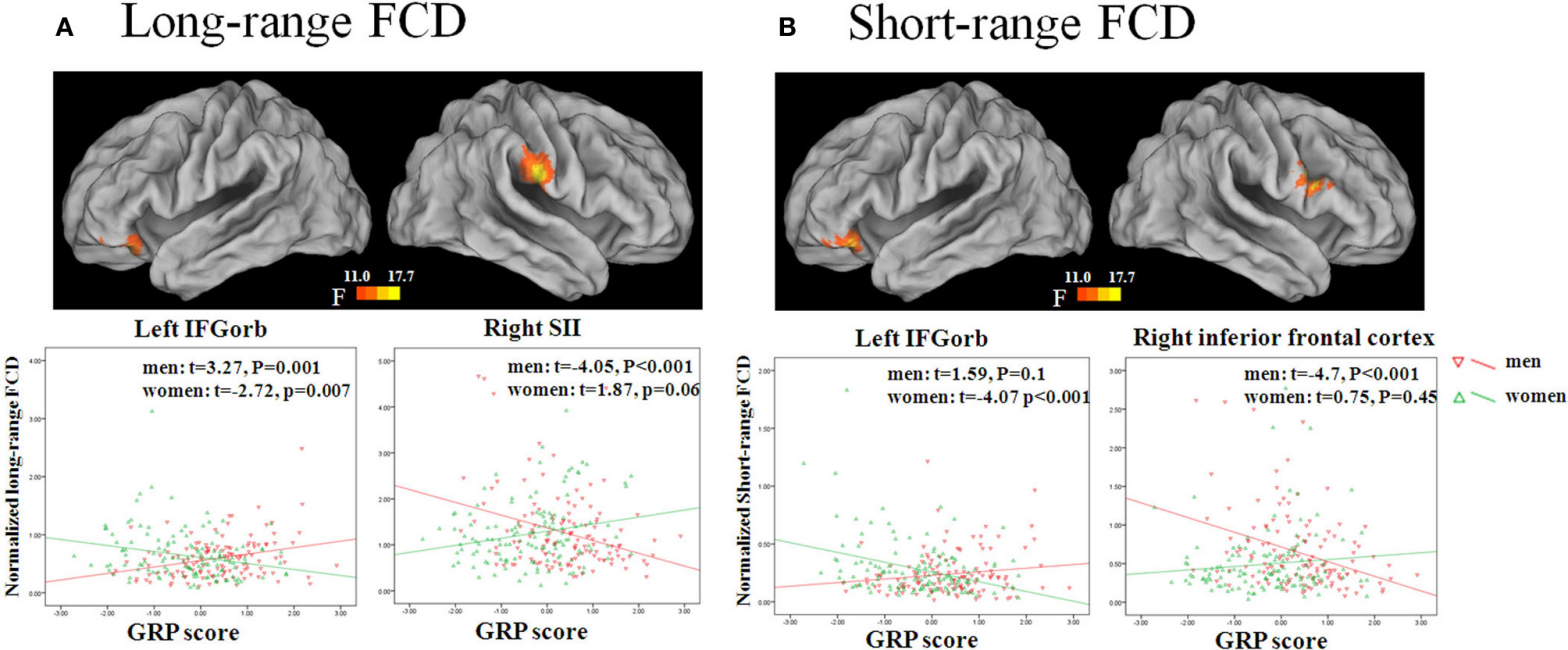
**Sex by GRP interaction effect for long-range FCD (A) and short-range FCD (B)**. The images were created using the CARET 5.62. Scatter plots show GRP-related changes in long- or short-range FCD in the male and female group. The lines are linear fits of the data.

The GRP by sex interaction was significant for long-range FCDs in the left inferior OFC and right supramarginal gyrus/postcentral gyrus (SII). Independent analyses of the average FCD values in the two regions validated the interaction effects [for the left inferior OFC (*β* = 0.25, *p* < 0.001), for the right SII (*β* = −0.25, *p* < 0.001)]. *Post-hoc* tests of these effects showed that the long-range FCD in the left inferior OFC was positively correlated with the GRP score for men (*β* = 0.11, *SE* = 0.03, *t* = 3.27, *p* = 0.0012), but negatively correlated with the GRP score for women (*β* = −0.08, *SE* = 0.03, *t* = −2.72, *p* = 0.007). Furthermore, the long-range FCD in the right SII was negatively correlated with the GRP score for men (*β* = −0.27, *SE* = 0.07, *t* = −4.05, *p* = 0.0001), but was unrelated for women (*β* = 0.11, 1.87, *p* = 0.06) (Figure 2, Table 3).

**Table 3:** **Sex by the general risk propensity score interaction effects on long- and short-range FCD**.

**Cluster size**	**Hemisphere**	**Brain region**	**BA**	**MNI coordinates**			**Peak *F*-value**
**LONG-RANGE FCD**							
68	Right	Supramarginal gyrus/postcentral gyrus	2/40	66	−18	24	19.42
30	Left	Inferior orbitofrontal cortex	11	−36	36	−15	18.96
**SHORT-RANGE FCD**							
27	Right	Inferior frontal gyrus	44/45	57	12	24	17.6
22	Left	Inferior orbitofrontal cortex	11	−42	36	−18	17.66

*Abbreviations: BA, broadman area; FCD, functional connectivity density*.

The GRP by sex interaction was also significant for short-range FCDs in the left inferior OFC and right IFG. Independent analysis of the average FCD values in the two regions also validated the interaction effects [for the left inferior OFC (*β* = 0.22, *p* < 0.001), for the right IFG (*β* = −0.23, *p* < 0.001)]. *Post-hoc* tests of these effects showed that the short-range FCD in the left inferior OFC was negatively correlated with the GRP score for women (*β* = −0.07, *SE* = 0.02, *t* = −4.07, *p* = 0.0001), but not for men (*β* = 0.03, *SE* = 0.02, *t* = 1.59, *p* = 0.1), and that the short-range FCD in the right IFG was negatively correlated with the GRP score for men (*β* = −0.19, *SE* = 0.04, *t* = −4.7, *p* < 0.0001), but not for women (*β* = 0.03, *SE* = 0.04, *t* = 0.75, *p* = 0.45) (Figure 2, Table 3)^1^^,^ ^2^.

### RSFC networks

**Figure 3:**
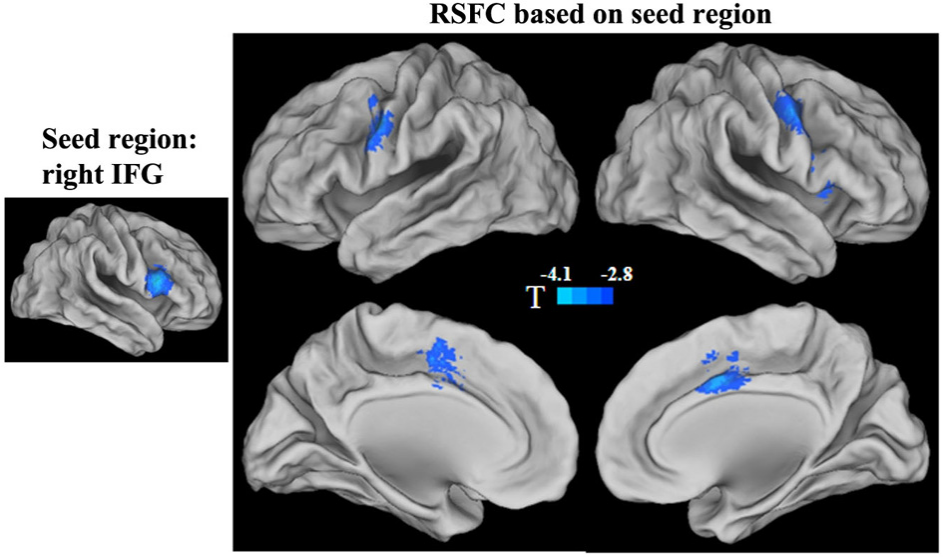
**Statistical significance (*T*-score) of the correlation with the GRP score for the strength of functional connectivities in the right IFG seed region**. The cool color represents a negative correlation.

Because both the short- and long-range FCD were correlated with GRP in the right IFG, we mapped the functional connectivity network of this region. We found that the RSFCs between the right IFG and the right anterior insula and the bilateral precentral gyrus/inferior prefrontal gyrus, as well as the middle cingulated cortex (MCC) and its adjacent supplementary motor cortex (SMA) were negatively correlated with the GRP score (*p* < 0.05, corrected) (Figure 3, Table 4).

**Table 4:** **Main effect of the general risk propensity scores on the RSFC in the right IFG seed region**.

**Cluster size**	**Hemisphere**	**Brain region**	**BA**	**MNI coordinates**			**Peak *F*-value**
50	Right	Anterior insular	13/45	45	21	6	16.57
46	Left	Precentral gyrus/IFG	6/9	−57	3	36	13.52
62	Right	Precentral gyrus/IFG	6/9	51	3	30	11.14
57	Bilateral	MCC/SMA	32/24/6	3	3	45	10.8

*Abbreviations: RSFC, resting-state functional connectivity; IFG, inferior frontal gyrus; MCC/SMA, the middle cingulated cortex and its adjacent supplementary motor cortex*.

**Figure 4:**
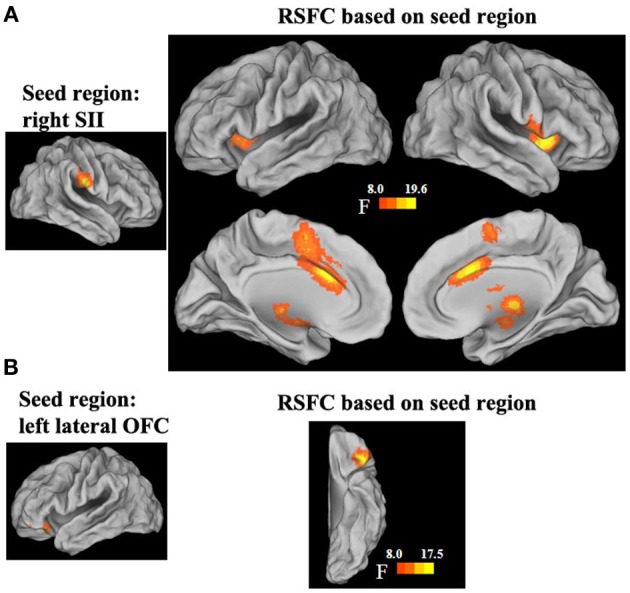
**Sex by GRP interaction effect in the strength of the functional connectivities of the right SII (A) and left lateral OFC. **(B)** seed region**.

The functional connectivity networks of the regions that showed a significant interaction effect in short- and long-range FCDs were also mapped. Significant interaction effects were found in the strength of the functional connectivities between the right SII and the bilateral insula extending to the striatum and thalamus, dorsal anterior cingulate cortex (dACC) and its adjacent medial prefrontal cortex, as well as the left cerebellar posterior lobe (*p* < 0.05, corrected). *Post-hoc* tests of the interaction effects showed that the strength of these functional connectivities was negatively correlated with the GRP for men (all *β* ≥ 0.04, *SE* ≥ 0.01, *t* ≤ −3.97, *p* < 0.001), but was unrelated for women (all *β* < 0.02, *SE* ≥ 0.01, *t* < 1.1, *p* > 0.05) (Figure 4, Table 5). Significant interaction effects were also found in the strength of the functional connectivities between the left inferior OFC and adjacent cortex. *Post-hoc* tests of the interaction effect showed that the strength of this functional connectivity was positively correlated with the GRP for men (*β* = 0.03, *SE* = 0.01, *t* = 2.64, *p* = 0.009), but was negatively correlated for women (*β* = −0.03, *SE* = 0.01, *t* = −3.03, *p* = 0.003).

**Table 5:** **Sex by the general risk propensity score interaction effects on the RSFC in the right SII seed region**.

**Cluster size**	**Hemisphere**	**Brain region**	**BA**	**MNI coordinates**			**Peak *F*-value**
750	Right	Insula/striatum/thalamus	13	39	12	−3	19.63
300	Bilateral	Dorsal anterior cingulate cortex/medial prefrontal cortex	32/24/6	3	18	33	18.07
251	Left	Insula/striatum/thalamus	13	−30	15	0	15.03
144	Left	Cerebellum posterior lobe		−30	−45	−45	14.22

*Abbreviations: RSFC, resting-state functional connectivity; SII, supramarginal gyrus/postcentral gyrus*.

## Discussion

Previous studies have emphasized the utility of RSFC methods in the investigation of inter-individual differences in brain function associated with enduring behavioral traits and tendencies (Di Martino et al., 2009; Cox et al., 2010; Hoptman et al., 2010; Adelstein et al., 2011). In the present study, we examined the neural correlates of the trait of GRP in resting-state fMRI in men and women using the recently developed FCDM method combined with seed-based RSFC. Our results revealed the existence of both shared and distinct functional networks associated with risk propensity in men and women.

### RSFC reliably measures intrinsic functional architecture and sex differences

In the current study, using FCDM, we identified the posterior cingulate/ventral precuneus and medial prefrontal cortices as the core regions in the intrinsic functional architecture, consistent with previous studies (Buckner et al., 2009; Tomasi and Volkow, 2010, 2012b; Zuo et al., 2012). We also found a significant sex difference for both short- and long-range FCDs. Women showed higher levels of short- and long-range FCDs in the default mode network regions, and lower levels of short- and long-range FCDs in the ventral frontal cortex and insula. This result is consistent with previous findings that the organization of intrinsic brain activity differs between males and females (Biswal et al., 2010; Zuo et al., 2010; Tomasi and Volkow, 2012b,c,d; Zuo et al., 2012; Filippi et al., 2013). The similarity of these results suggests that resting-state functional network analysis can provide a reliable measure for the neural correlates of the trait of GRP and its sex differences.

### Sex differences in the neural correlates of general risk propensity

In the current study, the interaction between sex and GRP was found in short- and/or long-range FCDs in the right secondary somatosensory cortex (SII) and the left OFC, suggesting that the resting-state neural correlates of risk propensity may differ between men and women. Seed-based RSFC further revealed the detailed functional connectivities associated with sex differences in the GRP.

A local network centered at the left inferior OFC was found to be differentially correlated with GRP in men and in women. This finding is consistent with previous task-based fMRI studies in which the inferior lateral OFC has also been identified as contributing to sex-related differences in risk-taking behavior (Bolla et al., 2004; Lee et al., 2009). In addition, we found that women have higher spontaneous activities within a local network centered at the left lateral OFC coupled with lower risk propensity. This is consistent with Lee et al's study, in which activity in the lateral OFC has been found to correlate negatively with the rate of selecting risky choices in women (Lee et al., 2009). However, these previous studies focused on the differences in the task-induced activity of the lateral OFC between men and women. The current study based on resting-state functional connectivity suggests that the functional connectivities within the lateral OFC also contributed to sex differences in GRP. Therefore, the results of the current study lend further support to a sex-related modulation of neural activity in the lateral OFC and extend this effect to spontaneous neural activity during rest.

A resting-state network centered at the right SII was also differentially associated with GRP in men and in women. This network included the bilateral insula extending to the striatum and thalamus, dACC, and left cerebellum. Increasing anatomical evidence has shown that extensive connections exist between the SII and thalamus (Krubitzer and Kaas, 1992; Qi et al., 2002), between the thalamus, dorsal anterior insula and dACC (Mufson and Mesulam, 1984; Vogt et al., 1987), and between the SII and posterior insula (Mesulam and Mufson, 1982; Mufson and Mesulam, 1982). These anatomical connections may be the structural basis of the resting-state network centered at the right SII. Although the role of the SII cortex in risk processing remains unclear, most of the component regions in this functional network have been implicated in risk processing based on a quantitative coordinate-based meta-analysis of studies that investigated the neural representations of risk (Mohr et al., 2010). Specifically, the anterior insula and thalamus have been identified as being involved in emotional risk processing and the dACC has been identified as involved in cognitive risk processing (Mohr et al., 2010). Our finding that the resting-state functional connectivities between the SII and these regions are differentially associated with GRP in men and in women suggests that future task-based fMRI studies should focus on the role of the SII in risk processing.

It is noteworthy that the dorsal anterior insula and the dACC, together with some cortical and subcortical structures such as thalamus, constitute a functional network which has been suggested as being involved in detecting the salience of external and internal stimuli, whether cognitive, homeostatic, or emotional, and prompting appropriate behavioral responses (Dosenbach et al., 2007; Seeley et al., 2007; Menon and Uddin, 2010; Legrain et al., 2011; Beissner et al., 2013). The unique functions identified in this network point to the possibility that the differential processing of salience information may determine at least some sex differences in GRP. Future research may shed some light on the nature of this possible connection.

### Shared neural correlates of general risk propensity in both men and women

For both men and women, GRP was linked with short- and long-range FCD in the right IFG, indicating that the stronger the resting-state functional connectivity of the right IFG, the lower the risk propensity. Task-induced response studies have shown that activity in the right IFG is related to risk aversion (Christopoulos et al., 2009; D'Acremont et al., 2009), consistent with lesion (Clark et al., 2003) and resting EEG (Gianotti et al., 2009) studies. Our results are consistent with these studies. Using seed-based RSFC, we further found a negative correlation between GRP and the strength of functional connectivity between the right IFG and right anterior insula. This is consistent with the results of a previous resting-state fMRI study in which risk-aversion was associated with stronger positive functional connectivity between the right IFG and the right insula (Cox et al., 2010). Our results not only replicate this previous finding but also reveal that the strength of the functional connectivity between the right IFG and MCC covaries with GRP. The MCC, which is located close to the dACC where we found sex differences in GRP, is also named as dACC in some studies (such as Dosenbach et al., 2007; Seeley et al., 2007; Menon and Uddin, 2010). Considering that both the MCC and right anterior insula are the core regions in a functional network (Dosenbach et al., 2007; Seeley et al., 2007; Menon and Uddin, 2010; Legrain et al., 2011; Beissner et al., 2013), these two regions may play similar roles in risk propensity.

We also found that short-range FCDs in the right dorsolateral prefrontal cortex, in addition to bilateral inferior frontal gyri and adjacent anterior insula, were negatively correlated with GRP. The dorsolateral prefrontal cortex is a core region in the frontoparietal component of the control system in the resting brain (Dosenbach et al., 2007, 2008). Previous studies have supported its role in risky decision making: transient suppression of activity in the right dorsolateral prefrontal cortex increases risk-taking behavior (Knoch et al., 2006), while upregulation of activity in this region decreases risk-taking behavior (Fecteau et al., 2007a,b). Our finding is consistent with these previous studies, and suggests that the functional connectivities within the dorsolateral prefrontal cortex are also related to GRP in both men and women.

### Several issues need to be addressed

The present study is based on the development of a composite measure of the trait of GRP. Generally there are two different views on measuring risk propensity (Harrison et al., 2005). On one view, risk taking is a function of a decision maker's risk perception and willingness to assume these perceived risks for an expected return. Choices across different risk domains therefore depend on enduring individual differences in risk perception and preferences. The alternative view is that risk propensity is an unstable trait across decision risk domains (such as finance, health/safety, social and so on) (such as Weber et al., 2002). Accordingly, there are two approaches to measure risk propensity. One approach, used here, is to identify relatively stable and domain-free traits associated with risk taking, with the traits of sensation seeking and impulsivity seen as particularly important (Harrison et al., 2005). The other approach is to measure separate aspects of risk propensity across multiple domains. In the present study, we have assumed that risk propensity, measured by the first method, is a stable individual trait and likely to be reflected in differences in resting-state brain functional activity. This is supported by the fact that our findings on the shared neural correlates of GRP in both men and women are consistent with a previous RSFC study (Cox et al., 2010), which similarly used healthy adults as participants.

In the present study, we used a whole brain search strategy based on FCDM combined with seed-based RSFC to examine the neural correlates of the GRP in men and women. One of the merits of this method is that it can identify the functional networks underlying risk propensity and its sex differences. This is an advance compared to the previous task-based fMRI studies (Bolla et al., 2004; Lee et al., 2009), which focused on activation of individual regions during risk-taking tasks. Because an understanding of how the human brain produces cognition and complex behaviors ultimately depends on the knowledge of a large scale brain organization (Bressler and Menon, 2010), the current findings improve our understanding of the neural basis of sex differences in risk propensity. Although it is difficult to reach a firm conclusion regarding the specific functional role of each region or network found by resting-state fMRI *per se* due to the lack of a directly involved task, the correspondence between regions identified by the two methods suggests some form of connection (Smith et al., 2009). Following a Hebbian view of experience dependent synaptic enhancement (Hebb, 1949), the correspondence between the regions identified by the current resting-state study and previous task-based studies suggests that the differences and similarities between men and women in the neural correlates of GRP may, at least in part, reflect a longstanding history of coactivation of specific brain regions as a network involved in the differential processing of risk in men and women. However, the exact nature of this connection awaits further research.

## Conclusions

The present study investigated the relationship between the enduring trait of GRP and intrinsic functional connectivity networks in men and women. We found patterns of sex differences that may underlie potential qualitative differences in the processing of risk by men and women. This work provides a new perspective on the brain-behavioral relationships in risky decision making and contributes to our understanding of sex differences in risk propensity.

## Author contributions

All authors were involved in the design and implementation of the study and the writing of the manuscript. Authors Yuan Zhou, Shu Li, Chunshui Yu, and Tianzi Jiang devised the concept and supervised the study. Authors Huandong Li, Wen Qin, and Maohu Zhu collected the imaging data. Authors Yuan Zhou, Huandong Li, Maohu Zhu, and Li-Lin Rao carried out the analysis. Authors Shu Li, John Dunn, Chunshui Yu, and Tianzi Jiang joined in the interpretation of data.

## Conflict of interest statement

The authors declare that the research was conducted in the absence of any commercial or financial relationships that could be construed as a potential conflict of interest.

## Figures and Tables

**Figure A3:**
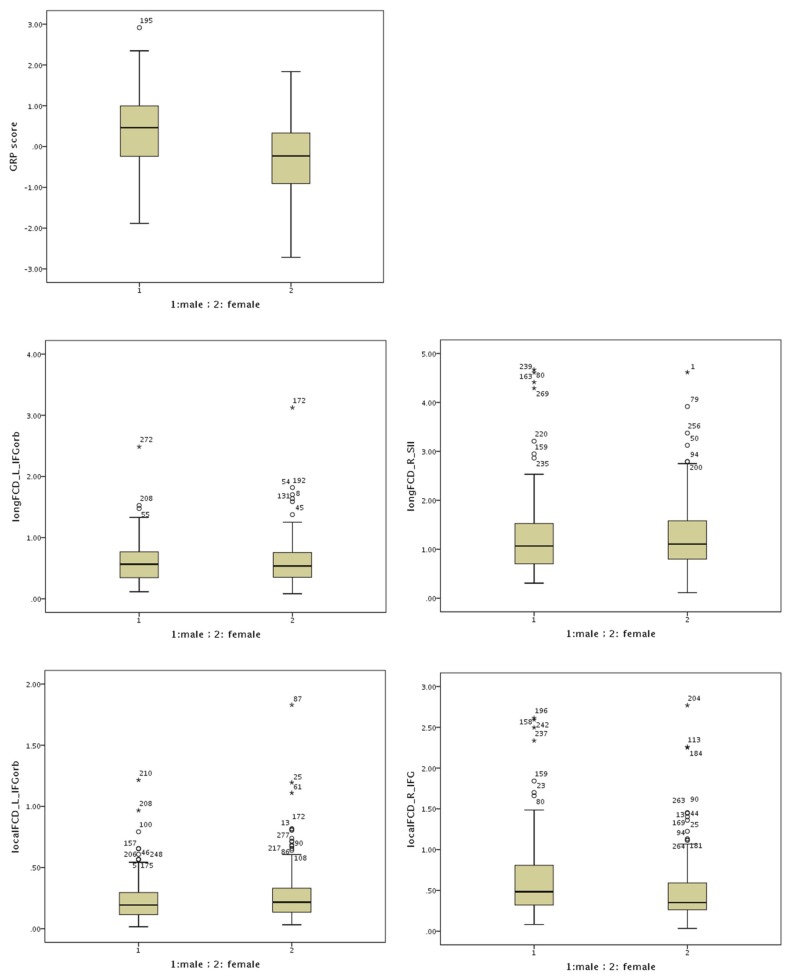
**Boxplots for the GRP score and the functional connectivity density (FCD) in males and females**. Open dots indicate a mild outlier with scores more than 1.5 * IQR from the rest of the scores. Stars indicate an extreme outlier with scores more than 3 * IQR from the rest of the scores. IQR stands for interquartile range and is the middle 50% of the scores.

## Footnotes

^1^As one of the reviewers noticed, there were some extreme outliners in the functional density measures Figure A3 in Appendix). When we re-analyzed the data after excluding these extreme outliers, we found that the GRP by sex interaction remained significant for long-range FCDs in the left inferior OFC (*β* = 0.22, *p* < 0.001) and right SII (*β* = −0.20, *p* = 0.001). The GRP by sex interaction also remained significant for short-range FCDs in the left inferior OFC (*β* = 0.15, *p* = 0.01) and right IFG (*β* = −0.18, *p* = 0.002).

^2^We acknowledge that the observed effect size is small, as one of the reviewers noticed. This is to be expected given that the low signal to noise ratio is inherent in resting state analyses. In order to test whether or not the interaction effect is robust, we randomly selected half of the original data set to determine whether or not the interaction effect remains significant. We found that the GRP by sex interaction remained significant for long-range FCDs in the left OFC (*β* = 0.27, *p* = 0.002) and the right SII (*β* = −0.33, *p* = 0.000). The GRP by gender interaction was also significant for short-range FCDs in the left inferior OFC (*β* = 0.21, *p* = 0.009) and right IFG (*β* = −0.22, *p* = 0.01).

## Appendix

**Table A1:** **Descriptive statistics of the assessment of risky propensity**.

	**Mean**	***SD***
RPS	3.2	1.2
BIS_ATT	16.2	3.1
BIS_MOT	21.7	3.6
BIS_NP	25.3	4.2
SSS_TAS	4.6	2.1
SSS_ES	3.1	1.7
SSS_DIS	4.1	2.0
SSS_BS	3.0	1.6
TPQ_NS	14.0	5.1
TPQ_HA	14.9	6.4
TPQ_RD	19.0	3.5
EPQ_P	4.4	2.8
EPQ_E	12.3	5.0
EPQ_N	10.9	5.4
EPQ_L	9.8	3.5

*Abbreviations: BIS_ATT, attentional factors of the Barratt Impulsiveness Scale; BIS_MOT, motor factors of the Barratt Impulsiveness Scale; BIS_NP, non-planning impulsiveness factors of the Barratt Impulsiveness Scale; EPQ_E, extraversion-introversion subscale of the Eysenck Personality Questionnaire; EPQ_N, neuroticism subscale of the Eysenck Personality Questionnaire; EPQ_P, psychotism subscale of the Eysenck Personality Questionnaire; RPS, Risk Propensity Scale; SSS_TAS, thrill and adventure seeking dimention of the Sensation Seeking Scale; SSS_ES, experience seeking dimention of the Sensation Seeking Scale; SSS_DIS, disinhibition dimention of the Sensation Seeking Scale; SSS_BS, boredom susceptibility dimention of the Sensation Seeking Scale; TPQ_NS, novelty seeking subscale of the Tridimensional Personality Questionnaire; TPQ_HA, harm avoidance subscale of the Tridimensional Personality Questionnaire; TPQ_RD, reward dependence subscale of the Tridimensional Personality Questionnaire*.
